# Novel biomineralization strategy in calcareous foraminifera

**DOI:** 10.1038/s41598-018-28400-2

**Published:** 2018-07-05

**Authors:** C. Borrelli, G. Panieri, T. M. Dahl, K. Neufeld

**Affiliations:** 1 Department of Earth and Environmental Sciences, University of Rochester, Rochester, NY 14627 USA; 2 CAGE - Centre for Arctic Gas Hydrate, Environment and Climate, Department of Geosciences, UiT The Arctic University of Norway in Tromsø, N-9037 Tromsø, Norway; 3 Department of Geosciences, UiT The Arctic University of Norway in Tromsø, N-9037 Tromsø, Norway

## Abstract

This work shows that calcareous benthic foraminifera are capable of agglutinating sedimentary particles also. In particular, we focus on *Melonis barleeanus*. Traditionally considered a calcareous species, our data revealed the presence of minute (~3 μm) sedimentary particles (silicate grains) inside the chamber walls of the examined shells. These particles were arranged in a definitive and systematic pattern, and the similar grain chemical characterization and size suggested a relatively high degree of selectivity in both modern and fossil specimens. Based on these results, we propose that *M*. *barleeanus* is capable of agglutinating sedimentary particles during the formation of a new chamber. The analysis of other calcareous foraminiferal species (e.g., *Cassidulina neoteretis*, *Lobatula lobatula*, *Nonionella stella*) did not reveal the presence of silicate grains in the shell of the specimens analyzed confirming this to be a characteristic of *M*. *barleeanus*. Considering that the isotopic and chemical composition of this species is widely used in paleoclimatic and paleoceanographic reconstructions, we used a mixing model to better constrain the influence of sedimentary particles on *M*. *barleeanus* δ^18^O data. Our model showed that the calcite δ^18^O would increase by ~0.9–2‰ if 10 wt% of feldspars (i.e., anorthite, albite, orthoclase) and quartz, respectively, were included in the analyzed shell. Based on these results, we emphasize that it is of paramount importance to consider *M*. *barleeanus* unusual biomineralization strategy during the interpretation of geological records and to investigate the presence of similar processes in other calcareous foraminiferal species.

## Introduction

All foraminifera are characterized by the presence of a shell (defined as “test” in foraminifera) that encloses the organism and separates it from the surrounding environment^1^. Such shell can be organic, agglutinated, or made of minerals precipitated by the foraminifer itself^1^. Among foraminifera, species that precipitate a shell of calcium carbonate (i.e., calcite or aragonite; CaCO_3_) are of particular importance because of their roles in the global carbon cycle and in paleoceanographic and paleoclimatic reconstructions^2–7^. Even so, the mechanisms behind the foraminiferal shell precipitation process (biomineralization) are not fully understood, yet^8,9^. In fact, there are many biological controls (overall defined as “vital effects”)^10^ that contribute to the shell precipitation in foraminifera, like the development of a delimited space where formation of a new chamber occurs^11,12^, production of organic sheets^12,13^, seawater endocytosis^14,15^, the presence of ion transporters^9,16^, and mitochondrial activity^17^. Considering that calcifying foraminifera are characterized by a variety of ecological and physiological differences, including the precipitation of a shell that can be calcitic or aragonitic, it is very likely that different species utilize diverse biomineralization processes to build their shell^15^, as suggested by the range of Mg concentrations measured among foraminiferal species^18^.

Calcareous foraminifera are a significant component of the global carbon cycle. For example, planktonic foraminiferal shells contribute to ~32–80% (0.36–0.88 Gt CaCO_3_ yr^−1^) of the calcite budget in deep oceanic settings^2^, whereas benthic foraminifera represent ~5% (0.03 Gt CaCO_3_ yr^−1^) of the accumulated CaCO_3_ in global reef sediments^19^. Even if biomineralization represents a sink for carbon, it is also a potential source of CO_2_ to the ocean-atmosphere system^20^, according to the simplified reaction:

$${{\rm{Ca}}}^{2+}+2{{\rm{HCO}}}_{3}^{-}={{\rm{CaCO}}}_{3}+{{\rm{CO}}}_{2}+{{\rm{H}}}_{2}{\rm{O}}$$

In calcareous foraminifera, the growth rate is influenced by the pH of the surrounding waters^16^. Therefore, the current increase in atmospheric CO_2_ concentration, and the consequent ocean acidification, is expected to impact foraminiferal biomineralization in a way that it is difficult to predict^21^. In addition, in seawater CaCO_3_ precipitation and dissolution are influenced by the saturation state of CaCO_3_ (Ω), which is function of [Ca^2+^], [CO_3_^2−^], and the CaCO_3_ stoichiometric solubility product at *in situ* temperature, pressure, and salinity. Thus, also changes in Ω can impact foraminiferal shell precipitation^22^. Finally, several studies demonstrated the influence of additional environmental parameters, like temperature, salinity, food, pollution among others, on foraminiferal biomineralization processes and shell morphology^23–25^.

Considering the contribution of calcareous foraminifera to the global carbon cycle and their importance as geological archives^8^, it is fundamental to explore the possibly species-specific biomineralization processes in these organisms. Vital effects, ecological preferences (e.g., habitat), and environmental parameters (e.g., temperature, pH) can all influence the chemical and isotopic composition of foraminiferal shells, causing some deviations from the expected isotopic and chemical equilibrium fractionations as defined by laboratory experiments involving the inorganic precipitation of CaCO_3_^8,9,18,26^. Interestingly, previous studies reported the presence of sedimentary grains within the calcareous shell of some benthic and planktonic foraminifera^27–29^. This aspect has to be considered and further investigated, at least in those species used to reconstruct changes in ocean circulation and climate through geological time. In fact, data collected using calcareous foraminiferal shells containing exogenous grains might be biased by the isotopic and chemical composition of such grains if their presence is unrecognized or unknown. This risk was already demonstrated for planktonic foraminifera, where the presence of silicate grains contaminated the Li isotope measurement conducted using an ion probe^29^.

In this study, we focus on *Melonis barleeanus*, a paleoceanographically-relevant calcareous benthic foraminiferal species^7^. In particular, we present a unique and vast microscopy and spectroscopy dataset collected analysing modern and fossil samples from the Arctic Ocean and Mediterranean Sea. Our data show that *M*. *barleeanus*, a calcareous species, is also capable of agglutinating sedimentary particles within its calcareous shell.

## Results

### Scanning electron microscopy (SEM) analysis of benthic foraminiferal species

**Figure 1:**
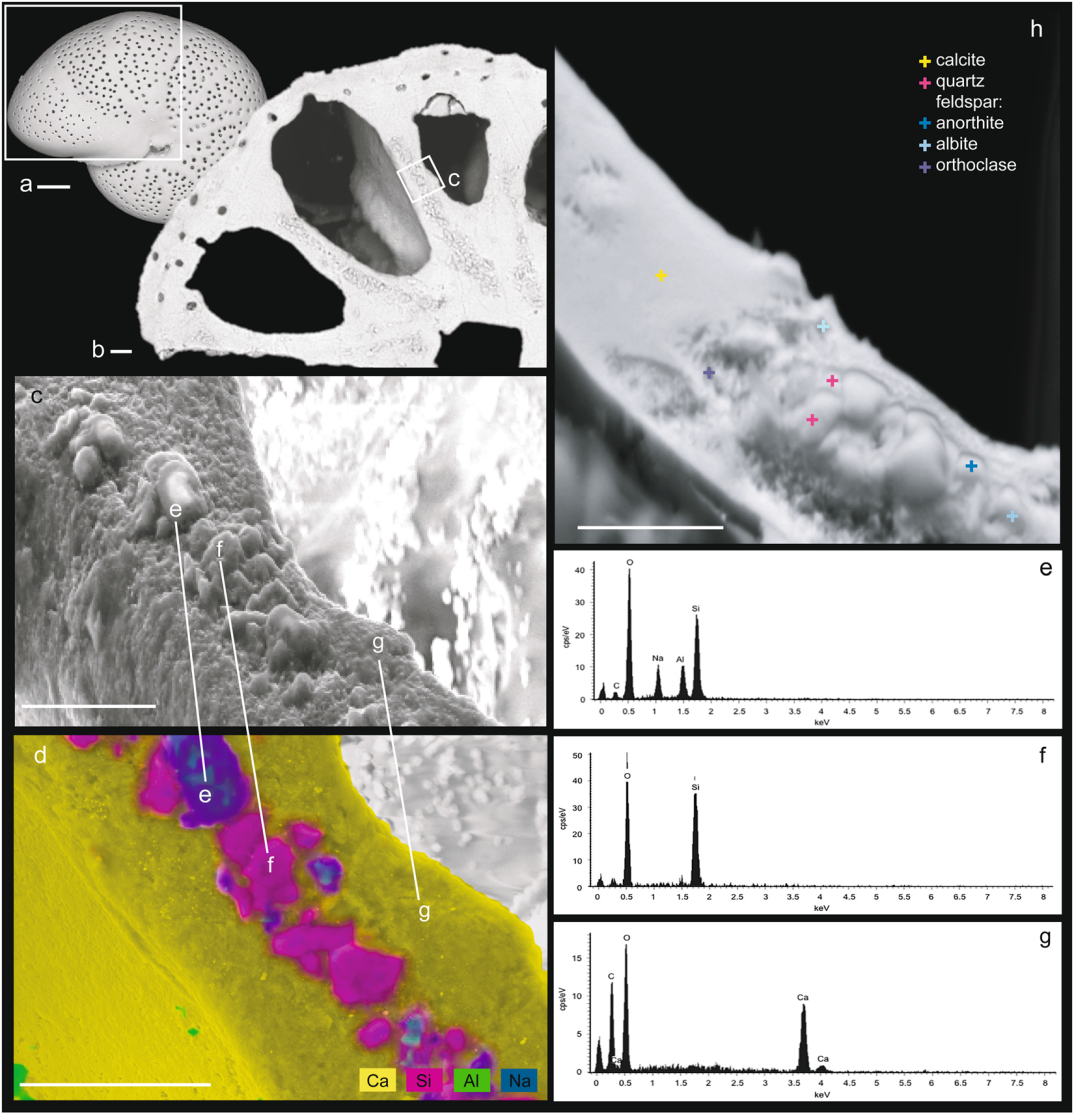
Distribution, chemical, and mineralogical characterization of sedimentary particles within the calcite shell of *Melonis barleeanus*. Micrograph (**a**) of the benthic foraminifer *M*. *barleeanus* (CAGE 15-2 893B 0–1 cm). White square indicates the backscatter electron (BSE) image from cross sections (**b**) where minute (~3 μm) sedimentary particles appear to be well organized inside the chamber wall of the foraminiferal shell. The secondary electron images show the grains (**c**) and their chemical characterization (**d**) presented as point analyses (**e**–**g** with relative spectra) and as compositional map (yellow indicates calcium, Ca; pink indicates silicon, Si; green indicates aluminum, Al; blue indicates sodium, Na). The electron backscattered diffraction image (**h**) is an overlaid image of backscatter electron image and forescatter electron image showing the phase identification via point analysis (colored crosses). Scale bars are 10 μm.

**Figure 2:**
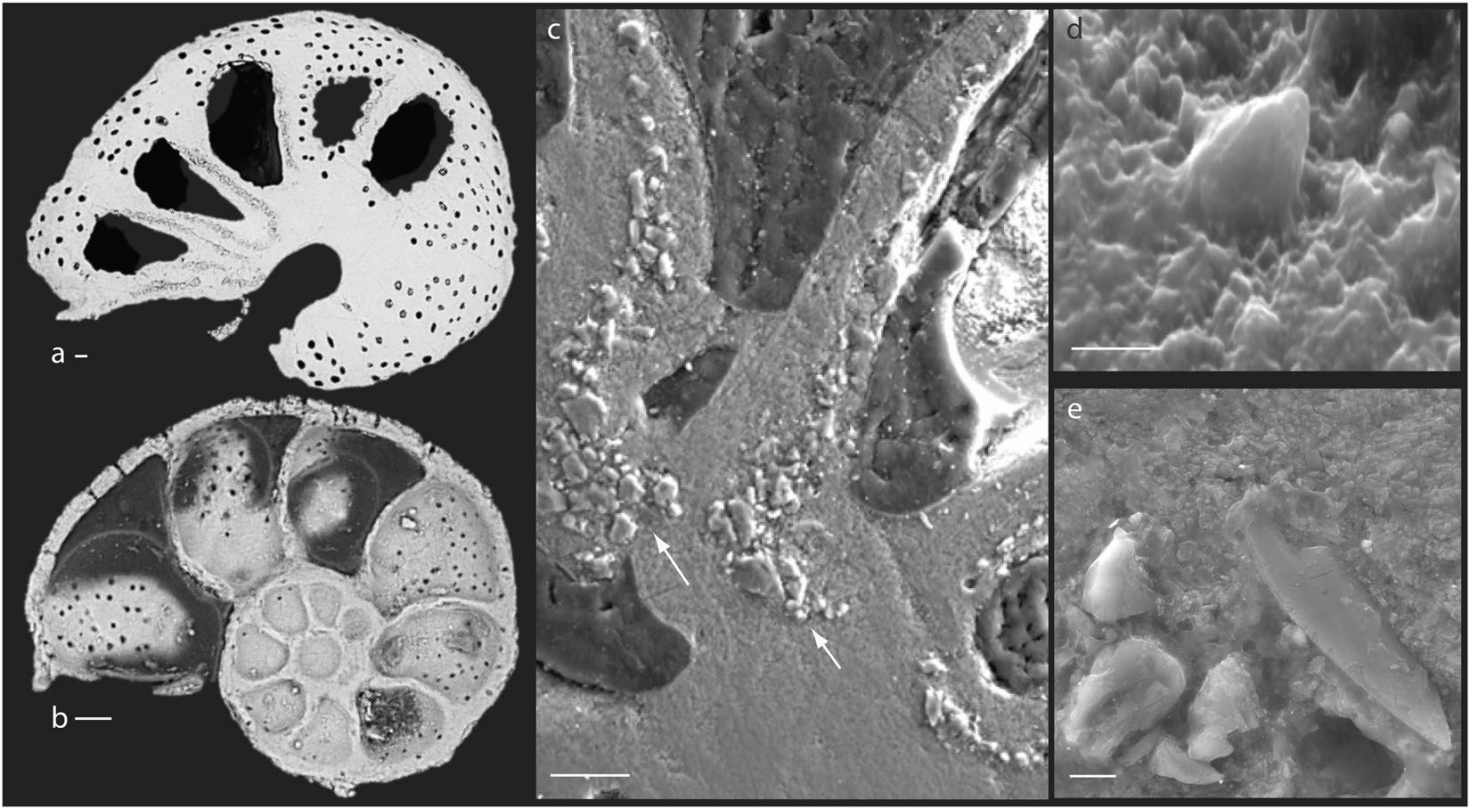
Sedimentary particle distribution within the calcite shell of *Melonis barleeanus*. (**a**,**b**) Backscatter electron image (BSE) of *M*. *barleeanus* specimens characterized by the presence (**a**) and absence (**b**) of sedimentary grains within the shell. In specimen (**a**) particles are linearly distributed within the wall between two chambers. (**c**) Cross-section of a shell containing several particles organized as groups (arrows) at the base of two chambers. (**d**) Minute sedimentary grain embedded in the shell calcite. (**e**) Broken diatom found within the shell of one *M*. *barleeanus* specimen. Scale bars are as follows: (**a**–**c**) 10 μm, (**d**,**e**) 1 μm.

We analysed 51 specimens from nine samples collected at different locations and time intervals (Tables 1 and 2; see Methods). Of the specimens examined, 61% showed the presence of minute sedimentary particles inside the chamber wall of the shell (Table 2; Figs 1 and 2). All the specimens with this feature (31 out of 51) belong to *Melonis barleeanus* (Williamson, 1858)^30^ (Figs 1a,b and 2a,b). None of the other species analysed (e.g., *Cassidulina neoteretis*, *Lobatula lobatula*, *Nonionella stella*) revealed a similar shell structure (Table 2).

**Table 1:** Summary of the cores used in this study. “N/a” = not applicable.

Core	Sampling device	Area	Water depth (m)	Coordinates
CAGE 15-2 880B	Multicorer	Western Svalbard, Arctic Ocean	889	78°41′26.9400″N; 8°14′30.0000″E
CAGE 15-2 893B	Multicorer	Western Svalbard, Arctic Ocean	1203	79°00′11.2799″N; 6°55′26.4359″E
HH13 000 BC	Boxcorer	Western Svalbard, Arctic Ocean	1207	78°58′49.9864″N; 7°03′44.3383″E
PC06	Pistoncorer	Western Svalbard, Arctic Ocean	374	78°36′39.6000″N; 9°25′31.8000″E
FR 320	Hammer	Monte Narbone Formation (Southern Sicily, Italy)	N/a	37°17′46.76″N; 13°27′15.63″E
VIB 10	Vibracorer	Gulf of Termini (Northern Sicily, Italy)	127	38°03′57.729″N; 13°39′57.8998″E

**Table 2:** Summary of the samples analyzed in this study.

Sample ID	Sample type	Species analyzed	# analyzed shells	# shells with sedimentary particles
CAGE 15-2 880B 0–1 cm	Modern	*M*. *barleeanus*	5 (5)	5
CAGE 15-2 880B 1–2 cm	Modern	*M*. *barleeanus*	4 (3)	3
CAGE 15-2 880B 1–2 cm	Modern	Multiple species^*^	10 (0)	0
CAGE 15-2 893B 0–1 cm	Modern	*M*. *barleeanus*	7 (7)	7
CAGE 15-2 893B 1–2 cm	Modern	*M*. *barleeanus*	9 (9)	9
HH13 000 BC 1–2 cm	Modern	*M*. *barleeanus*	2 (0)	2
PC06 5 34–35 cm	Fossil	*M*. *barleeanus*	1 (0)	1
FR 320	Fossil	*M*. *barleeanus*	5 (0)	1
VIB 10 138 cm 72–74 cmbsf	Fossil	*M*. *barleeanus*	8 (0)	3

The numbers among parentheses indicate the number of rose Bengal stained specimens examined. Cmbsf = cm below the sea floor. ^*^Eilohedra nipponica (Kuwano, 1962; 3 specimens), *Cibicidoides* sp. (Thalmann, 1939; 1 specimen), *Nonionella stella* (Cushman and Moyer, 1930; 1 specimen), *Lobatula lobatula* (Walker and Jacob, 1798; 1 specimen), *Cassidulina neoteretis* (Seidenkrantz, 1995; 3 specimens), and *Cassidulina reniforme* (Nørvang, 1945; 1 specimen).

Regardless of the sample origin (Arctic Ocean vs. Mediterranean Sea – Sicily) or age (modern vs. fossil), our data revealed that *M*. *barleeanus* was characterized by the presence of sedimentary particles within its calcite shell, although this was not a universal feature of this species. In fact, 24% of the *M*. *barleeanus* considered in this study did not have any grain embedded in their shell (Fig. 2b). When present, the spatial distribution of the sedimentary particles appeared to be well-organized and not random. More specifically, we found the grains to be arranged linearly, very often within the wall between two chambers (Figs 1b,c and 2a; Supplementary Table S1), in all the specimens analysed. These stretches of particles were between a few μm and several tens of μm long (Supplementary Table S2). The calcite layer on both sides of the grain stretches was found to be of variable thickness (Supplementary Table S2), even if this could be a consequence of the sample cross-section preparation. Some of the shell examined (19%) was characterized also by the presence of several particles organized as a group of a few tens of μm across. This group of grains was usually located at the base of one or more chambers (Fig. 2c; Supplementary Tables S1 and S2). In general, the silicate grains were disposed very close to each other regardless of their spatial organization (linear stretch vs. group; Figs 1b,c and 2a,c). Less frequently, grains were separated by a gap of ~1 to 10 μm (Supplementary Table S2). We noted that in a few cases some grains were arranged close to pores, which were generally visible only on the exterior of the shell and rarely within the chamber wall (Supplementary Table S2), although this might be the result of the type of section prepared (cross-section). Laminated calcite (secondary calcite)^8^ was identified on the edge of the shell of a few specimens (Supplementary Table S2).

In order to test for the possibility that the silicate grains observed in many of the *M*. *barleeanus* specimens examined could derive from the protocol used to prepare the foraminiferal samples prior analysis (i.e., polishing with Al_2_O_3_ powder and colloidal silica), several *M*. *barleeanus* specimens were analysed after the shell was mechanically fractured, but not embedded in epoxy and polished. Also in this case, the presence of silicate grains was recognized, confirming that the results of this study were not biased by the sample preparation protocol used.

### Energy-dispersive X-ray spectroscopy (EDS) and electron backscattered diffraction (EBSD) analyses of silicate grains

Three hundred grains were analysed for size (see Methods). A high degree of variability was noted in the size of the grain incorporated in the shells, which ranged from 0.8 μm to 10.2 μm (average = 2.8 μm, SD = 1.4 μm) (Supplementary Table S3). Based on the visible portion of the grains, the layer containing sedimentary particles was at least 1–5 μm thick (Figs 1c and 2d). In general, particles were organized as a linear stretch and/or group regardless of size (Supplementary Tables S1–S3). No significant relationship was found between the grain size and the foraminiferal size neither in modern nor in fossil samples, independently from their geographic origin. Of the grains analysed for size, we examined 273 of them through energy-dispersive X-ray spectroscopy (EDS) (see Methods; Supplementary Table S3). Based on the semi-quantitative EDS chemical characterization (see Methods), we classified the particles analysed as quartz-like (grains composed of O and Si; Figs. 1d–g), which represented 49% of the particles examined (Supplementary Table S3), and feldspar-like (grains composed of O, Si, and Al and/or Ca and/or Na; Figs. 1d–g), which represented 29% of the particles analysed (Supplementary Table S3). This classification was confirmed by electron backscattered diffraction (EBSD) of selected grains (see Methods; Fig. 1h; Supplementary Fig. S1). Finally, 22% of the examined particles was not classified at all because the low spatial resolution of the EDS maps prevented unambiguous characterization of the grain chemical makeup.

Foraminifera from the Mediterranean Sea were mostly characterized by quartz-like particles compared to feldspar-like ones (55% vs. 13%, respectively; number of grains analysed = 53, of which 32% could not be unequivocally classified) (Supplementary Table S3). This finding applied to the samples from the Arctic Ocean, as well even if the difference in the grain distribution was smaller (47% quartz-like particles vs. 33% feldspar-like ones; number of grains analysed = 220, of which 20% could not be unequivocally classified) (Supplementary Table S3). It is possible that this diversity in grain distribution is a consequence of the different number of *M*. *barleeanus* specimens from the Mediterranean Sea vs. Arctic Ocean available for analysis (Table 2).

## Discussion

*Melonis barleeanus* is an infaunal species widely used in paleoceanographic studies^7,31,32^. Traditionally considered a calcareous species^33^, our data indicate that *M*. *barleeanus* is also characterized by the presence of silicate grains within its shell. Even if, to our knowledge, one extant foraminiferal order is capable of secreting opaline silica (i.e., Silicoloculinida)^34^, we do not think that the silicate grains found within many of the *M*. *barleeanus* examined were precipitated by the foraminifer itself. In fact, the particles observed vary in their chemical makeup, size, and shape, thus making it highly unlikely that a single foraminiferal species could have been able to precipitate such a variety of grains. Instead, we think that it is more likely that the grains originated from the specimens’ surroundings and were agglutinated by the organism. Analysis of the sediment fraction <63 μm of a sample collected close to the foraminiferal sampling sites in the Arctic Ocean revealed the presence of quartz, feldspars, clay minerals, and mica, supporting this hypothesis. This hypothesis was also supported by the finding of a diatom frustule within the shell of one of the specimens investigated (Fig. 2e).

The systematic spatial arrangement of the grains within the shell of both modern and fossil foraminifera (Figs 1b,c and 2a,c) indicates that the presence of sedimentary particles in *M*. *barleeanus* is not a simple case of contamination or diagenesis. The presence of sedimentary particles within the shell of rotaliid species was already reported in a few benthic foraminiferal genera (i.e., *Cibicides* and *Stomatorbina*)^27,28^. In these foraminifera, grains were arranged as an almost continuous layer within the septal wall (*Cibicides*)^27^ or were contained within the aragonite material of the inner primary wall (*Stomatorbina*)^28^, a spatial distribution similar to the one we observed in *M*. *barleeanus*. Sedimentary particles were also reported for planktonic foraminifera (e.g., *Globigerinoides*)^29^. Even if a clear explanation for the presence of agglutinated grains in benthic foraminifera was not provided, it was proposed that the particles were not secreted by the foraminifer and were not contaminants^35^, a suggestion that agrees with our findings. In planktonic foraminifera, the presence of alumino-silicate grains within the shells analysed was explained as the consequence of calcite precipitation from seawater vacuoles containing small silicate grains^29^.

In foraminifera, seawater vacuolization^8,15,29^ is one of the processes involved in the formation of a new chamber. Even if this process was invoked to explain the presence of minute particles within the shell of planktonic species^29^, we do not think that seawater vacuolization is a satisfactory explanation of our results. In fact, our data showed that not all the *M*. *barleeanus* analysed were characterized by the presence of sedimentary particles. In addition, the investigation of additional specimens belonging to both epifaunal (e.g., *Lobatula lobatula*) and shallow infaunal (e.g., *Cassidulina neoteretis*) species did not reveal the presence of silicate grains within the shells examined (Table 2). If seawater vacuoles containing small sedimentary particles were responsible for our observations, then we would have expected silicate grains in all the specimens analysed, or at least in all the infaunal ones. Experiments involving *Ammonia tepida* demonstrated that vacuolized seawater was used as a source of Ca^2+^ and inorganic carbon involved in the calcification process of this species, but that seawater was not directly released at the extracellular calcification site^15^. Although it is uncertain if and to what extent the mineralization processes observed in one foraminiferal species can be applied to another one^15^, we think that seawater vacuoles might not be directly released at the calcification site in *M*. *barleeanus*, as well. In this context, seawater endocytosis cannot explain the presence (or absence) of sedimentary particle within the shell of this species. Additional studies, possibly involving culture experiments, are necessary to better constrain the role of seawater vacuoles during the chamber formation in *M*. *barleeanus*.

The presence of silicate grains within the shell of *M*. *barleeanus* might be explained by the accidental incorporation of foreign particles (e.g., detritus, sediment, algal cells, sponge spicules) forming a secondary shell (i.e., feeding cyst) surrounding the foraminifer’s original shell^36,37^. Laboratory observations showed that *M*. *barleeanus* was mostly free of a secondary shell when in the sediment^37^. However, one of the specimens analysed revealed the presence of a diatom among the particles incorporated (Fig. 2e), supporting the hypothesis that this species might incorporate some grains/detritus forming the secondary shell during the formation of a new chamber. Unfortunately, the data available do not allow us to draw a firm conclusion about this hypothesis. As an alternative, incorporation of sedimentary particles might occur also during the formation of a “delimited space” prior chamber formation^8^. In this case, it is possible that silicate grains surrounding the calcifying foraminifer might interact with the organic layer that defines the shape of the new chamber and that promotes crystal nucleation. According to this scenario, sedimentary particle simply adhere to the organic template and are incorporated during precipitation of the chamber wall. Even if this is a reasonable explanation of our data, we think that it is still an unsatisfactory one. In fact, the formation of a space that isolates the foraminifer from the surrounding environment is a common step during biomineralization process in perforate foraminifera^8^. Even so, we do not observe the presence of silicate grains in all the *M*. *barleeanus* specimens analysed and in the other perforate foraminiferal species included in this study (Table 2).

Our data showed that *M*. *barleeanus* preferred particles with a size comprised between 0.8 and 10.2 μm and with a quartz-like mineralogy (Supplementary Table S3). It is possible that the mineralogy of the sedimentary particles incorporated by this foraminiferal species reflect the diverse sediment composition at the Mediterranean Sea vs. Arctic Ocean sampling sites. However, considering that in the Arctic Ocean sediment analysed as part of this study the quartz content varied between 30 and 40% (vs. 47% as measured in the *M*. *barleeanus* specimens from the Arctic Ocean), we think that this species has a distinct preference for quartz-like sedimentary particles compared to feldspar-like ones (Supplementary Table S3). At this stage, we cannot draw a firm conclusion about the reason(s) of this preference, but we note that selectivity towards a particular mineralogy was demonstrated already for several species of agglutinated foraminifera^38,39^ and it was proposed for the miliolid species *Rudoloculina hooperi*^40^, as well. Based on our results and noting that the silicate grains are mostly associated with *M*. *barleeanus* primary calcite^8^, we propose that this species agglutinates sedimentary particles prior the formation of a new chamber.

If agglutination prior calcite precipitation is a strategy to promote calcite precipitation and/or to increase the test strength is unclear at this stage. Crystal nucleation on a pre-existing surface (heterogeneous nucleation) is energetically more advantageous compared to homogeneous crystal nucleation^41^; however, in foraminifera, the organic matrix (or primary organic sheet) represents the nucleation site for the first CaCO_3_ crystals^8^. Based on the compatibility of the calcite and quartz crystal lattices, it is unclear if precipitation of calcite on a quartz surface is possible^42^ or not^43^, but laboratory experiments showed that the presence of quartz seeds induced calcium carbonate nucleation in the bulk solution in unstable supersaturated solutions (domain of spontaneous precipitation)^42^. Considering the location of the sedimentary particles within *M*. *barleeanus* (mostly within the chamber wall), we think that it is more likely that silicate grains were incorporated to enhance the shell mechanical strength. Increased mechanical strength through agglutination was already hypothesized for some agglutinated foraminifera^44^.

Many conditions can affect shell precipitation in foraminifera, like pH, temperature, salinity, food, pollution, and environmental energy among others^21–25^. We note that nearby Arctic sites CAGE 15-2 880B and CAGE 15-2 893B (~900 m and 1200 m water depth, respectively), hereafter referred to as Core 880B MC and Core 893B MC, the temperature was 0.69 °C and 0.77 °C, salinity was 34.89‰ and 34.86‰, and pH ranged from 8.021 to 8.257. According to these data, we do not think that environmental conditions at the sampling sites represented an impediment to foraminiferal biomineralization.

The genes involved in the foraminiferal biomineralization processes are still unknown. Therefore, we can only speculate that the (facultative) agglutinated strategy adopted by some *M*. *barleeanus* specimens might be genetically controlled. The analysis of the complete small subunit ribosomal RNA gene (SSU rDNA) demonstrated that rotaliids and textulariids (*Trochammina* sp. and *Eggerelloides scabrum*) were closely related^45^. Therefore, it might be possible that some of the genes involved in the shell formation of agglutinated (textulariid) species might still be present in the genome of rotaliid species and that the expression of these genes might be triggered by conditions that remain uncertain at the moment. Interestingly, it was proposed that environmental conditions could have influenced the biomineralization pathway of the genus *Miliammina*^46^. A genetically controlled agglutinating biomineralization strategy can definitely explain why *M*. *barleeanus*, but also *Cibicides floridanus*^27^ and *Stomatorbina*^28^, are characterized by the presence of agglutinated particles within their shell.

Foraminifera are an important component of the global carbon cycle because of their role in the oceanic food webs^47^ and calcium carbonate precipitation^2,19^. Therefore, the effect that ocean acidification will have on this group of organisms is of a great interest. Published studies involving culture experiments and the investigation of natural samples^21^ showed that different foraminiferal species respond differently (from decalcification to higher calcification rates)^21^ to decreasing ocean pH values. In this context, the biomineralization strategy adopted by *M*. *barleeanus* provides a new insight regarding the strategies adopted by these unicellular organisms to build their shells. Published studies identified the presence of sedimentary particles within the shell of other calcareous foraminiferal species^27–29^, suggesting that agglutination prior (or during) calcification might not be a characteristic of *M*. *barleeanus* specimens only, even if it is possible that the presence of sedimentary particles within the shell of different calcareous foraminifera is a consequence of species-specific calcification pathways (see also^29^).

*M*. *barleeanus* is an important benthic species that is often used in paleoclimatic and paleoceanographic reconstructions^7,31,32^. In the light of the results of this study, we think that it is of fundamental importance to consider the *M*. *barleeanus* biomineralization strategy during the interpretation of geological records. Contamination of isotopic (or chemical) measurements due to the presence of silicate grains is a likely possibility, in particular when the presence of exogenous grains is not identified prior *in-situ* analysis (e.g., electron probe, ion probe, laser ablation). This risk was already demonstrated for Li isotope measurements conducted on planktonic foraminifera with an ion probe^29^. In this study, we used a mixing model to investigate the influence of sedimentary particles on δ^18^O data collected using *M*. *barleeanus* “contaminated” by the presence of silicate grains (Supplementary Fig. S2). Our calculations showed that the δ^18^O of a hypothetical pure calcite end-member would increase by ~0.9–2‰ if 10 wt% of feldspars (i.e., anorthite, albite, orthoclase) and quartz, respectively, were included in the analyzed calcite (Supplementary Fig. S2). The analytical (vs. theoretical) quantification of the influence of sedimentary particles on the isotopic data collected using “contaminated” *M*. *barleeanus* is going to be the focus of a follow-up study.

Future microscopy and spectroscopy studies using other benthic and planktonic foraminiferal species will be critical to gain a better insight about how frequently sedimentary particles can be found within the shell of paleoceanographically relevant foraminiferal species. In addition, culture-based experiments and molecular studies will help to better understand if the presence of silicate grains within the shell of benthic and planktonic foraminifera is a genetically-driven mechanism and what are the conditions that can trigger such unusual biomineralization strategy.

## Methods

### Foraminiferal sample preparation and data collection

Samples locations are listed in Table 1. Modern samples (Table 2) were preserved in a solution of deionized water and rose Bengal and stored at 4 °C. Samples were not sieved and the picking was performed directly on the wet sediment. Fossil samples (Table 2) were oven-dried first and then wet sieved at 63 μm. The residual fraction was oven-dried again prior foraminiferal picking. None of the foraminiferal specimens analysed for this study showed signs of dissolution.

Modern (recently dead and rose Bengal stained) and fossil foraminifera (Table 2) were treated following the same published protocol^48^. First, foraminifera were ultrasonically cleaned for a few seconds, so to remove any residual clays prior analysis. Organic matter was then removed as follows: (1) 2 rinses with ultrapure H_2_O; (2) 3 oxidative steps with hot buffered hydrogen peroxide (1:1 0.1 N extra pure NaOH, Acros Organics, and 30% low trace metals H_2_O_2_, Veritas) held at 70 °C for 30 minutes each step; (3) 5 rinses with ultrapure H_2_O. Finally, samples were leached 2 times with 0.001 N trace metal grade HNO_3_ (Fisher Scientific) followed by 2 final rinses in ultrapure H_2_O^48^. At the end of the procedure, several foraminifera showed few rose Bengal stained spots. Because of this, an additional oxidative step was performed, followed by 5 rinses with ultrapure H_2_O.

Samples were mounted in epoxy (EpoxyCure®, Buehler), and let dry overnight at room temperature. The epoxy mount was polished using 800 and 1200 grit silicon carbide papers (Buehler) and 1 μm Al_2_O_3_ powder (Mark V Laboratories) to expose the internal structure of each foraminiferal shell. In addition, several *M*. *barleeanus* specimens were mechanically broken on the longitudinal plane. Without any prior treatment, shell fragments were first inspected with a scanning electron microscope (SEM) Hitachi Tabletop Microscope TM-3000 (The Arctic University of Norway in Tromsø, Tromsø, Norway). Then, the fragments were carbon-coated and analysed with a SEM Zeiss Merlin VP Compact (The Arctic University of Norway in Tromsø, Tromsø, Norway).

For preparation of sample PC06 5 34–35 cm see^49^.

Scanning electron microscopy and energy-dispersive X-ray spectroscopy (EDS) were performed at The Arctic University of Norway in Tromsø (Tromsø, Norway) using an SEM Hitachi Tabletop Microscope TM-3000 equipped with a Bruker Quantax 70 EDS Detector. Measurements of several parameters (e.g., grain size, grain stretch length, thickness of calcite layer on both sides of the particle stretch; Supplementary Tables S2 and S3) were conducted analysing SEM pictures with the software ImageJ^50^. Prior measurement of the selected parameter(s), a scale was set using the scale bar associated with every picture analysed and the possibility to distinguish grain boundary was carefully evaluated. Reported grain size refers to the particle maximum diameter (Supplementary Table S3).

EDS chemical maps were generated for every *M*. *barleeanus* specimen characterized by the presence of sedimentary particles. These maps were visually inspected for qualitative analyses of element distribution within the studied material and phase identification. To distinguish the foraminiferal calcite from the sedimentary particles, we focused on the presence of aluminium (Al), calcium (Ca), oxygen (O), silicon (Si), and sodium (Na) in the material analysed. Data are reported in Supplementary Table S3 and were used in the calculations regarding grain distribution. Quartz-like grains were distinguished based on the presence of O and Si in the particles. Feldspar-like grains were identified based on the presence of O, Si, and Al and/or Ca and/or Na. Selected *M*. *barleeanus* specimens from Core 880B MC and Core 893B MC were also analysed with a SEM Zeiss Merlin VP Compact equipped with an EDS x-max 80 system by Oxford instruments, combined with the analytical software AZtec (The Arctic University of Norway in Tromsø, Tromsø, Norway). These samples were gold-coated prior analysis. These EDS data for chemical characterization are presented as point analyses and as a compositional map in Fig. 1. A list of the EDS conditions used in this study is given in Supplementary Table S4.

Phase identification via point analysis was conducted by electron backscattered diffraction (EBSD) using the SEM Zeiss Merlin VP Compact (The Arctic University of Norway in Tromsø, Tromsø, Norway) equipped with a NordlysNano EBSD detector from Oxford instruments. The AZtec software was used for data acquisition and processing into an EBSD point map. The electron image used for the EBSD point map is a layered image from backscatter and forescatter imaging. For EBSD analysis, the manually polished epoxy mount was chemically polished with a colloidal silica solution (OP-U, Struers) as the detector takes its signal from the uppermost 50 nm of the sample surface. The sample was tilted to an angle of 70°. A list including the EBSD conditions used in this study and the detected phases is given in the Supplementary Table S4.

### Sediment sample analysis

A sediment sample from the Arctic Ocean was analysed to characterize the average mineralogical composition of the sediment in the area where Cores 880B MC and 893B MC were collected. In order to do so, the sediment fraction <63 μm was dried, mounted on carbon tape, and carbon-coated. EDS analysis of the sample were conducted using a SEM Zeiss Merlin VP Compact equipped with an EDS x-max 80 system by Oxford instruments, combined with the analytical software AZtec (The Arctic University of Norway in Tromsø, Tromsø, Norway).

### Environmental conditions at Core 880B MC and Core 893B MC

To further investigate the possibility of a relationship between the presence of sedimentary particles within the shell of *M*. *barleeanus* and the environment in which the foraminifera calcified their shell, we collected several environmental parameters in proximity of Core 880B MC and Core 893B MC. These two sites were chosen because of the availability of environmental data and “living” (rose Bengal stained) *M*. *barleeanus* specimens for analysis (Table 2).

In proximity of Core 880B MC and Core 893B MC, temperature and salinity were measured with a CTD (Conductivity Temperature Depth) SBE 9 plus sensor, whereas pH was measured with a SBE25plus 1049. Close to the seafloor, temperature was 0.69 °C and 0.77 °C and salinity was 34.89‰ and 34.86‰ for locations near sites Core 880B MC and Core 893B MC, respectively. pH data were available from nearby stations and ranged from 8.021 to 8.257.

All data generated or analysed during this study are included in this published article (and its Supplementary Information files).

## Electronic supplementary material

Supplementary Information

## References

[1] Goldstein, S. T. In *Modern Foraminifera* (ed Sen Gupta, B. K.) 37–56 (Kluwer Academic Press, 2002).

[2] Schiebel R (2002). Planktic foraminiferal sedimentation and the marine calcite budget. Global Biogeochem. Cy..

[3] Cramer BS, Toggweiler JR, Wright JD, Katz ME, Miller KG (2009). Ocean overturning since the Late Cretaceous: Inferences from a new benthic foraminiferal isotope compilation. Paleoceanography.

[4] Hönisch B, Hemming NG, Archer D, Siddall M, McManus JF (2009). Atmospheric carbon dioxide concentration across the mid-Pleistocene transition. Science.

[5] Coxall HK, Wilson PA (2011). Early Oligocene glaciation and productivity in the eastern equatorial Pacific: Insights into the carbon cycling. Paleoceanography.

[6] Borrelli C, Cramer BS, Katz ME (2014). Bipolar Atlantic deepwater circulation in the middle-late Eocene: effects of Southern Ocean gateway openings. Paleoceanography.

[7] Panieri G, Graves CA, James RH (2016). Paleo-methane emissions recorded in foraminifera near the landward limit of the gas hydrate stability zone offshore western Svalbard. Geochem. Geophy. Geosy..

[8] Erez, J. In *Biomineralization* Vol. 54 (eds Dove, P. M. *et al*.) 115–149 (The Mineralogical Society of America, 2003).

[9] de Nooijer LJ, Spero HJ, Erez J, Bijma J, Reichart GJ (2014). Biomineralization in perforate foraminifera. Earth-Sci. Rev..

[10] Weiner, S. & Dove, P. M. In *Biomineralization* Vol. 54 (eds Dove, P. M. *et al*.) 1–29 (The Mineralogical Society of America, 2003).

[11] Angell RB (1980). Test morphogenesis (chamber formation) in the foraminiferal *Spiroloculina hyalina* Schulze. J. Foram. Res..

[12] Hemleben, C., Anderson, O. R., Berthold, W. & Spindler, M. In *Biomineralization in lower plants and animals* Vol. 30 (eds Leadbeater, B. S. *et al*.) 237–249 (Clarendon Press, 1986).

[13] Bé AWH, Hemleben C, Anderson OR, Spindler M (1979). Chamber formation in planktic foraminifera. Micropaleontology.

[14] Bentov, S., Brownlee, C. & Erez, J. The role of seawater endocytosis in the biomineralization process in calcareous foraminifera. *Proc*. *Natl*. *Acad*. *Sci*. *USA* **106**, 21500–21504, 10.1073_pnas.0906636106 (2009). 10.1073/pnas.0906636106 PMC2799886 20007770

[15] de Nooijer LJ, Langer G, Nehrke G, Bijma J (2009). Physiological controls on seawater uptake and calcification in the benthic foraminiferal *Ammonia tepida*. Biogeosciences.

[16] Ter Kuile B, Erez J, Padan E (1989). Mechanisms for the uptake of inorganic carbon by two species of symbiont-bearing foraminifera. Mar. Biol..

[17] Spero H (1988). Ultrastructural examination of chamber morphogenesis and biomineralization in the planktonic foraminifer *Orbulina universa*. Mar. Biol..

[18] Bentov S, Erez J (2006). Impact of biomineralization processes on the Mg content of foraminiferal shells: a biological perspective. Geochem. Geophy. Geosy..

[19] Langer MR, Silk MT, Lipps JH (1997). Global ocean carbonate and carbon dioxide production: the role of reef foraminifera. J. Foramin, Res..

[20] Barker S, Jiggins JA, Elderfield H (2003). The future of the carbon cycle: review, calcification response, ballast and feedback on atmospheric CO_2_. Phil. Trans. R. Soc. Lond. A..

[21] Keul N, Langer G, de Nooijer LJ, Bijma J (2013). Effect of ocean acidification on the benthic foraminifera *Ammonia* sp. is caused by a decrease in carbonate ion concentration. Biogeosciences.

[22] Haynert, K. & Schönfeld, J. Impact of changing carbonate chemistry, temperature, and salinity on growth and test degradation of the benthic foraminifer. *Ammonia aomoriensis*. *J*. *Foramin*. *Res*. **44**(2), 76–89, 10/2113/gsfjr.44.2.76 (2014).

[23] Boltovskoy E, Scott DB, Medioli FS (1991). Morphological variations of benthic foraminiferal tests in response to changes in ecological parameters: a review. J. Paleont..

[24] Dissard D, Nehrke G, Reichart GJ, Bijma J (2010). The impact of salinity on the Mg/Ca and Sr/Ca ratio in the benthic foraminifera *Ammonia tepida*: results from culture experiments. Geochim. Cosmochim. Acta.

[25] Barras C, Geslin E, Duplessy J-C, Jorissen FJ (2009). Reproduction and growth of the deep-sea benthic foraminifer *Bulimina marginata* under different laboratory conditions. J. Foramin. Res..

[26] Katz M (2010). Traditional and emerging geochemical proxies in foraminifera. J. Foramin. Res..

[27] Bellamo S (1976). Wall ultrastructure in the foraminifer *Cibicides floridanus* (Cushman). Micropaleontology.

[28] Hofker J (1978). Biological results of the SnelliusExpedition XXX: the foraminifera collected in 1929 and 1930 in the eastern part of the Indonesian Archipelago. Zoologische Verhandelingen.

[29] Vigier N, Rollion-Bard C, Spezzaferri S, Brunet F (2007). *In situ* measurements of Li isotopes in foraminifera. Geochem. Geophy. Geosy..

[30] Williamson, W. C. *On the recent foraminifera of Great Britain* (Ray Society, 1858).

[31] Koho KA, de Nooijer LJ, Reichart GJ (2015). Combining benthic foraminiferal ecology and shell Mn/Ca to deconvolve past bottom water oxygenation and paleoproductivity. Geochim. Cosmochim. Acta.

[32] Hasenfratz AP (2017). Mg/Ca-temperature calibration for the benthic foraminifera *Melonis barleeanum* and *Melonis pompilioides*. Geochim. Cosmochim. Acta.

[33] Sen Gupta, B. K. In *Modern Foraminifera* (ed. Sen Gupta, B. K.) 7–36 (Kluwer Academic Press, 2002).

[34] Resig, J. M., Lowenstam, H. A., Echols, R. J. & Weiner, S. In *Studies in marine micropaleontology and paleoecology*, *a memorial volume to Orville R*. *Bandy* Vol. 29 (ed. Sliter, W. V.) 205–214 (Allen Press Inc., 1980).

[35] Hansen, H. J. In *Modern Foraminifera* (ed. Sen Gupta, B. K.) 57–70 (Kluwer Academic Press, 2002).

[36] Cedhagen T (1996). Foraminiferans as food for cephalaspideans (Gastropoda: Opisthobranchia), with notes on secondary tests around calcareous foraminiferans. Phuket Marine Biological Center Special Publication.

[37] Heinz P, Geslin E, Hemleben C (2005). Laboratory observations of benthic foraminiferal cysts. Mar. Biol. Res..

[38] Heron-Allen E, Earland A (1912). On some foraminifera from the North Sea, etc., dredged by the fisheries cruiser “Goldseeker” (International North Sea Investigations — Scotland). I. On some Astrorhizidae and their shell-structure. J. R. Microsc. Soc..

[39] Jorgensen NO (1977). Wall structure of some arenaceous foraminifera from the Maastrichtian white chalk (Denmark). J. Foramin. Res..

[40] Guilbault J-P, Patterson RT (1998). *Rudolocilina hooperi*, a new miliolid with and agglutinated outer surface from the northeastern Pacific Ocean. J. Foramin, Res..

[41] De Yoreo, J. J. & Vekilov, P. G. In *Biomineralization* Vol. 54 (eds Dove, P. M. *et al*.) 57–93 (The Mineralogical Society of America, 2003).

[42] Lioliou MG, Paraskeva CA, Koutsoukos PG, Payatakes AC (2007). Heterogeneous nucleation and growth of calcium carbonate on calcite and quartz. J. Colloid. Interf. Sci..

[43] Lin Y-P, Singer PC (2005). Effects of seed material and solution composition on calcite precipitation. Geochim. Cosmochim. Acta.

[44] Lipps JH (1973). Test structure in foraminifera. Annu. Rev. Microbiol..

[45] Schweizer M, Pawlowski J, Kouwenhoven TJ, Guiard J, van der Zwaan B (2008). Molecular phylogeny of Rotaliida (Foraminifera) based on complete small subunit rDNA sequences. Mar. Micropaleontol..

[46] Habura A, Goldstein ST, Parfrey LW, Bowser SS (2006). Phylogeny and ultrastructure of *Miliammina fusca*: evidence for secondary loss of calcification in a miliolid foraminifer. J. Eukaryot. Microbiol..

[47] Gooday, A. J., Levin, L. A., Linke, P. & Heeger, T. In *Deep-sea food chains and the global carbon cycle* (eds Rowe G. T. *et al*.) 63–91 (Springer, 1992).

[48] Russell AD, Hönisch B, Spero HJ, Lea DW (2004). Effects of seawater carbonate ion concentration and temperature on shell U, Mg, and Sr in cultured planktonic foraminifera. Geochim. Cosmochim. Acta.

[49] Panieri G (2017). Diagenetic Mg-calcite overgrowths on foraminiferal tests in the vicinity of methane seeps. Earth Planet. Sc. Lett..

[50] Schneider CA, Rasband WS, Eliceiri KW (2012). NIH Image to ImageJ: 25 years of image analysis. Nat. Methods.

