# Supplementary material for: Novel biomineralization strategy in calcareous foraminifera
Source: Sci Rep. 2018 Jul 5;8:10201. doi: 10.1038/s41598-018-28400-2 (PMC6033919; doi:10.1038/s41598-018-28400-2)
Supplement: Supplementary file 1 — Supplementary Information [file 41598_2018_28400_MOESM1_ESM.docx]

**Supplementary Information**

**Novel biomineralization strategy in calcareous foraminifera**

C. Borrelli^1*^, G. Panieri^2^, T. M. Dahl^3^, and K. Neufeld^3^

^1^Department of Earth and Environmental Sciences, University of Rochester, Rochester, NY, 14627, USA

^2^ CAGE - Centre for Arctic Gas Hydrate, Environment and Climate, Department of Geosciences, UiT The Arctic University of Norway in Tromsø, N-9037, Tromsø, Norway

^3^ Department of Geosciences, UiT The Arctic University of Norway in Tromsø, N-9037, Tromsø, Norway

* Corresponding author: cborrelli@ur.rochester.edu

**Supplementary Table S1.** Summary of the spatial distribution analysis of silicate grains within *Melonis barleeanus* specimens. * Number of partially or fully exposed chambers.

| Specimen | # exposed chambers* | Silicate grains –  linear arrangement | Silicate grains – group |
| --- | --- | --- | --- |
| CAGE 15-2 880B 0-1 cm - Specimen 1 | 4 | Yes | Yes |
| CAGE 15-2 880B 0-1 cm - Specimen 2 | 2 | Yes | Yes |
| CAGE 15-2 880B 0-1 cm - Specimen 3 | 4 | Yes | No |
| CAGE 15-2 880B 0-1 cm - Specimen 4 | 3 | Yes | Yes |
| CAGE 15-2 880B 0-1 cm - Specimen 5 | 6 | Yes | Yes |
| CAGE 15-2 880B 1-2 cm - Specimen 6 | 5 | Yes | No |
| CAGE 15-2 880B 1-2 cm – Specimen 7 | 6 | Yes | No |
| CAGE 15-2 880B 1-2 cm - Specimen 8 | 6 | Yes | No |
| CAGE 15-2 893B 0-1 cm - Specimen 9 | 5 | Yes | No |
| CAGE 15-2 893B 0-1 cm - Specimen 10 | 6 | Yes | No |
| CAGE 15-2 893B 0-1 cm - Specimen 11 | 6 | Yes | No |
| CAGE 15-2 893B 0-1 cm - Specimen 12 | 4 | Yes | No |
| CAGE 15-2 893B 0-1 cm - Specimen 13 | 3 | Yes | No |
| CAGE 15-2 893B 0-1 cm - Specimen 14 | 2 | Yes | Yes |
| CAGE 15-2 893B 0-1 cm - Specimen 15 | 6 | Yes | No |
| CAGE 15-2 893B 1-2 cm - Specimen 16 | 3 | Yes | No |
| CAGE 15-2 893B 1-2 cm - Specimen 17 | 6 | Yes | No |
| CAGE 15-2 893B 1-2 cm - Specimen 18 | 6 | Yes | No |
| CAGE 15-2 893B 1-2 cm - Specimen 19 | 4 | Yes | No |
| CAGE 15-2 893B 1-2 cm - Specimen 20 | 7 | Yes | No |
| CAGE 15-2 893B 1-2 cm - Specimen 21 | 2 | Yes | No |
| CAGE 15-2 893B 1-2 cm - Specimen 22 | 3 | Yes | No |
| CAGE 15-2 893B 1-2 cm - Specimen 23 | 2 | Yes | No |
| CAGE 15-2 893B 1-2 cm - Specimen 24 | 7 | Yes | No |
| HH13 000BC 1-2 cm - Specimen 12 | 5 | Yes | No |
| HH13 000BC 1-2 cm - Specimen 13 | 5 | Yes | No |
| VIB 10 138 72-74 cm - Specimen 2 | 7 | Yes | Yes |
| VIB 10 138 72-74 cm - Specimen 3 | 6 | Yes | No |
| VIB 10 138 72-74 cm - Specimen 4 | 5 | Yes | No |
| FR 320 - Specimen 10 | 2 | Yes | No |
| PC06 Sect5 - Specimen 4 | 20 | Yes | No |

**Supplementary Table S2.** Characterization of the spatial distribution of silicate grains within *Melonis barleeanus* specimens. Measurements were conducted using the software ImageJ (see Methods). “N/a” = not applicable.

| **Specimen** | **Silicate grains – linear arrang. stretch length (μm)** | **CaCO_3_ thickness on both sides of the linear stretch (μm)** | **Silicate grains – group “diameter” (μm)** | **Gap between grains (μm)** | **Relationship grain size/ grain location** | **Proximity of grains to shell pores** | **Presence of pores in the chamber wall** | **Visible lamellar calcite** |
| --- | --- | --- | --- | --- | --- | --- | --- | --- |
| CAGE 15-2 880B 0-1 cm | 14.142 | 7.534-8.148 | 27.409 | 1.364 | No | No | No | No |
| Specimen 1 | 17.660 | 7.970-4.020 |  |  |  |  |  |  |
|  | 30.410 | 4.449-7.657 |  |  |  |  |  |  |
|  | 62.780 |  |  |  |  |  |  |  |
| CAGE 15-2 880B 0-1 cm | 14.622 | 2.951-4.883 | 19.069 | 2.406 | No | No | No | No |
| Specimen 2 | 28.748 | 6.074-1.650 |  | 3.777 |  |  |  |  |
|  |  |  |  | 1.774 |  |  |  |  |
| CAGE 15-2 880B 0-1 cm | 23.356 | 8.292-4.933 | na | 3.164 | No | No | No | No |
| Specimen 3 | 52.363 | 2.975-6.047 |  |  |  |  |  |  |
|  | 40.199 | 5.837-10.503 |  |  |  |  |  |  |
| CAGE 15-2 880B 0-1 cm | - | - | 11.877 | 1.132 | No | No | No | No |
| Specimen 4 |  |  |  |  |  |  |  |  |
| CAGE 15-2 880B 0-1 cm | 45.476 | 2.795-6.421 | 21.132 | - | More bigger | No | No | No |
| Specimen 5 | 57.958 | 7.514-4.832 |  |  | grains in the |  |  |  |
|  | 45.779 | 8.390-2.397 |  |  | central group |  |  |  |
| CAGE 15-2 880B 1-2 cm | - | - | na | - | - | Yes - few grains | No | No |
| Specimen 6 |  |  |  |  |  |  |  |  |
| CAGE 15-2 880B 1-2 cm | 16.938 | 3.294-2.988 | na | - | No | No | No | Yes |
| Specimen 7 |  |  |  |  |  |  |  |  |
| CAGE 15-2 880B 1-2 cm | 18.381 | 2.965-3.663 | na | 4.129 | No | Yes - few grains | No | No |
| Specimen 8 | 9.656 | 5.936-2.048 |  | 0.876 |  |  |  |  |
|  | 56.480 | 14.49-2.872 |  | 2.497 |  |  |  |  |
|  |  | 8.114-6.553 |  |  |  |  |  |  |
|  |  | 9.152-3.873 |  |  |  |  |  |  |
| CAGE 15-2 893B 0-1 cm | - | - | na | - | - | Yes - few grains | No | No |
| Specimen 9 |  |  |  |  |  |  |  |  |
| CAGE 15-2 893B 0-1 cm | 49.470 | 7.793-8.256 | na | 2.987 | No | Yes - few grains | No | No |
| Specimen 10 | 48.955 | 8.193-9.231 |  | 2.433 |  |  |  |  |
|  | 92.807 | 10.387-8.617 |  |  |  |  |  |  |
|  | 61.776 |  |  |  |  |  |  |  |
| CAGE 15-2 893B 0-1 cm | - | - | na | - | No | Yes - few grains | No | No |
| Specimen 11 |  |  |  |  |  |  |  |  |
| CAGE 15-2 893B 0-1 cm | - | - | na | - | No | Yes - few grains | No | No |
| Specimen 12 |  |  |  |  |  |  |  |  |
| CAGE 15-2 893B 0-1 cm | 42.546 | 8.981-3.198 | na | - | No | Yes - few grains | No | No |
| Specimen 13 | 22.720 | 5.788-10.834 |  |  |  |  |  |  |
| CAGE 15-2 893B 0-1 cm | 17.532 | - | 16.877 | 4.475 | No | No | No | No |
| Specimen 14 | 11.879 |  |  |  |  |  |  |  |
| CAGE 15-2 893B 0-1 cm | 16.131 | - | na | 2.450 | No | No | No | Yes |
| Specimen 15 |  |  |  |  |  |  |  |  |
| CAGE 15-2 893B 1-2 cm | - | - | na | 3.665 | No | Yes - few grains | One pore | No |
| Specimen 16 |  |  |  | 6.025 |  |  |  |  |
|  |  |  |  | 1.996 |  |  |  |  |
| CAGE 15-2 893B 1-2 cm | 20.492 | 3.590-7.084 | na | - | No | No | No | Yes |
| Specimen 17 | 12.705 | 5.520-4.500 |  |  |  |  |  |  |
|  | 20.722 |  |  |  |  |  |  |  |
| CAGE 15-2 893B 1-2 cm | - | - | na | - | No | No | No | No |
| Specimen 18 |  |  |  |  |  |  |  |  |
| CAGE 15-2 893B 1-2 cm | 19.650 | 7.752-4.467 | na | 2.083 | No | Yes - few grains | No | No |
| Specimen 19 | 14.923 |  |  | 8.723 |  |  |  |  |
| CAGE 15-2 893B 1-2 cm | - | - | na | - | No | No | No | No |
| Specimen 20 |  |  |  |  |  |  |  |  |
| CAGE 15-2 893B 1-2 cm | 31.868 | 9.422-8.533 | na | 2.126 | No | Yes - few grains | No | No |
| Specimen 21 | 14.560 |  |  | 4.919 |  |  |  |  |
|  | 66.364 |  |  |  |  |  |  |  |
| CAGE 15-2 893B 1-2 cm | - | - | na | - | More smaller grains in the | Yes - few grains | No | No |
| Specimen 22 |  |  |  |  | chamber wall |  |  |  |
| CAGE 15-2 893B 1-2 cm | 11.279 | 7.093-8.820 | na | - | No | Yes - few grains | No | No |
| Specimen 23 | 11.891 |  |  |  |  |  |  |  |
|  | 11.895 |  |  |  |  |  |  |  |
| CAGE 15-2 893B 1-2 cm | 66.795 | 7.005-4.526 | na | 5.082 | No | Yes - few grains | Few | Yes |
| Specimen 24 | 93.810 | 5.192-4.606 |  | 5.743 |  |  |  |  |
|  | 30.985 |  |  |  |  |  |  |  |
| HH13 000BC 1-2 cm | - | - | na | - | No | No | No | Yes |
| Specimen 12 |  |  |  |  |  |  |  |  |
| HH13 000BC 1-2 cm | - | - | na | - | No | No | Few | Yes |
| Specimen 13 |  |  |  |  |  |  |  |  |
| VIB 10 138 72-74 cm | 19.245 | - | 19.08 | 2.516 | No | Yes - few grains | No | No |
| Specimen 2 | 30.318 |  |  | 2.946 |  |  |  |  |
| VIB 10 138 72-74 cm | - | 4.307-5.222 | na | 2.522 | No | No | No | No |
| Specimen 3 |  |  |  | 1.708 |  |  |  |  |
|  |  |  |  | 1.832 |  |  |  |  |
| VIB 10 138 72-74 cm | - | - | na | - | No | No | No | No |
| Specimen 4 |  |  |  |  |  |  |  |  |
|  |  |  |  |  |  |  |  |  |
|  |  |  |  |  |  |  |  |  |
|  |  |  |  |  |  |  |  |  |
|  |  |  |  |  |  |  |  |  |
|  |  |  |  |  |  |  |  |  |
| FR 320 - Specimen 10 | 23.432 | - | na | 6.190 | No | Yes - few grains | No | No |
|  |  |  |  | 4.176 |  |  |  |  |
|  |  |  |  | 10.104 |  |  |  |  |
|  |  |  |  | 2.322 |  |  |  |  |
| PC06 Sect5 - Specimen 4 | - | - | na | - | No | Yes | No | No |

**Supplementary Table S3.** Summary of the size and chemical characterization analyses of silicate grains in *Melonis barleeanus* specimens. The average size of the grains was estimated using the software ImageJ (see Methods). The chemical characterization of the grains was extrapolated from visual inspection of energy-dispersive x-ray spectroscopy (EDS) maps. No point analyses are reported in this table. “x” is used for elements identified in the grain. “N/a” denotes sedimentary particles for which EDS data are not available. “?” indicates inconclusive results due to the spatial resolution of the maps (EDS aperture = 150 μm).

| Specimen | Grain analyzed | Grain size (μm) | Al | Ca | Na | O | Si |
| --- | --- | --- | --- | --- | --- | --- | --- |
| CAGE 15-2 880B 0-1 cm - Specimen 1 | 1 | 5.528 |  |  |  | x | x |
|  | 2 | 2.310 | ? | x |  | ? |  |
|  | 3 | 3.496 | x |  | x | x | x |
|  | 4 | 2.106 |  | x | x | x | x |
|  | 5 | 2.335 | x |  | x | x | x |
|  | 6 | 3.636 |  | x |  | x | x |
|  | 7 | 2.875 |  |  |  | x | x |
|  | 8 | 4.798 | x |  |  | x | x |
|  | 9 | 5.343 |  |  |  | x | x |
|  | 10 | 4.050 |  | x |  | x | x |
| CAGE 15-2 880B 0-1 cm - Specimen 2 | 1 | 3.912 | x |  | x | x | x |
|  | 2 | 2.975 |  |  |  | x | x |
|  | 3 | 4.170 |  |  |  | x | x |
|  | 4 | 2.578 |  |  |  | x | x |
|  | 5 | 3.579 | x |  | x | x | x |
|  | 6 | 1.684 |  | ? | x | x | x |
|  | 7 | 6.062 |  |  |  | x | x |
|  | 8 | 2.904 |  |  |  | x | x |
|  | 9 | 1.531 | n/a | n/a | n/a | n/a | n/a |
|  | 10 | 4.909 |  |  |  | x | x |
|  |  |  |  |  |  |  |  |
| CAGE 15-2 880B 0-1 cm - Specimen 3 | 1 | 4.995 | x |  | x | ? | ? |
|  | 2 | 3.391 |  |  |  | x | x |
|  | 3 | 2.550 | x |  | x | x | x |
|  | 4 | 2.800 |  |  |  | x | x |
|  | 5 | 2.340 | x |  | x | x | x |
|  | 6 | 1.686 |  | ? |  | x | x |
|  | 7 | 4.172 | x |  | x | x | x |
|  | 8 | 3.048 | x |  | x | x | x |
|  | 9 | 3.621 |  |  |  | x | x |
|  | 10 | 2.625 |  | ? |  | x | x |
| CAGE 15-2 880B 0-1 cm - Specimen 4 | 1 | 5.675 | x |  |  | x | x |
|  | 2 | 4.776 | x |  | x | x | x |
|  | 3 | 1.965 | x |  | x | x | x |
|  | 4 | 4.237 |  |  |  | x | x |
|  | 5 | 2.369 |  |  |  | x | x |
|  | 6 | 4.628 |  |  |  | x | x |
|  | 7 | 1.285 | x |  | x | x | x |
|  | 8 | 1.954 |  |  |  | x | x |
|  | 9 | 2.051 |  |  |  | x | x |
|  | 10 | 1.327 | x | x | x | x | ? |
| CAGE 15-2 880B 0-1 cm - Specimen 5 | 1 | 5.734 | n/a | n/a | n/a | n/a | n/a |
|  | 2 | 3.591 |  |  |  | x | x |
|  | 3 | 2.157 | n/a | n/a | n/a | n/a | n/a |
|  | 4 | 3.049 | n/a | n/a | n/a | n/a | n/a |
|  | 5 | 4.735 |  |  |  | x | x |
|  | 6 | 2.322 |  |  |  | x | x |
|  | 7 | 3.776 | x |  | x | x | x |
|  | 8 | 2.487 |  |  |  | x | x |
|  | 9 | 6.320 |  |  |  | x | x |
|  | 10 | 2.026 |  |  |  | x | x |
| CAGE 15-2 880B 1-2 cm - Specimen 6 | 1 | 3.549 |  | ? |  |  | ? |
|  | 2 | 3.262 |  |  |  | x | x |
|  | 3 | 1.901 |  |  | ? | x | x |
|  | 4 | 1.681 | x | x |  | x | x |
|  | 5 | 1.792 |  | x |  |  | x |
| CAGE 15-2 880B 1-2 cm - Specimen 7 | 1 | 1.911 |  | x |  |  |  |
|  | 2 | 1.537 |  |  |  | x | x |
|  | 3 | 1.396 | n/a | n/a | n/a | n/a | n/a |
|  | 4 | 3.261 |  |  |  | x | x |
| CAGE 15-2 880B 1-2 cm - Specimen 8 | 1 | 7.335 |  |  |  | x | x |
|  | 2 | 5.292 |  |  |  | x | x |
|  | 3 | 1.865 |  |  |  | x | x |
|  | 4 | 2.084 |  |  |  | x | x |
|  | 5 | 2.126 | x |  |  | ? | x |
|  | 6 | 4.261 | x |  |  | x | x |
|  | 7 | 4.246 | x |  | x | x | x |
|  | 8 | 1.701 | x | x | x | x | x |
|  | 9 | 2.498 |  | x |  | x | x |
|  | 10 | 1.701 | n/a | n/a | n/a | n/a | n/a |
| CAGE 15-2 893B 0-1 cm - Specimen 9 | 1 | 3.519 |  |  |  | x | x |
|  | 2 | 2.873 |  |  |  | x | x |
| CAGE 15-2 893B 0-1 cm - Specimen 10 | 1 | 8.648 |  |  |  | x | x |
|  | 2 | 6.177 |  |  |  | x | x |
|  | 3 | 2.046 |  |  |  | x | x |
|  | 4 | 2.418 | x |  | x | x | x |
|  | 5 | 4.444 | x | ? | x | ? | ? |
|  | 6 | 2.248 |  |  |  | x | x |
|  | 7 | 10.190 |  |  |  | x | x |
|  | 8 | 4.040 |  |  |  | x | x |
|  | 9 | 7.704 | x |  | x | x | x |
|  | 10 | 5.326 |  | ? |  | x | x |
| CAGE 15-2 893B 0-1 cm - Specimen 11 | 1 | 6.670 |  |  |  | x | x |
|  | 2 | 3.194 | x |  | x | x | x |
|  | 3 | 3.772 | x |  |  | x | x |
|  | 4 | 1.480 |  |  |  | x | x |
|  | 5 | 3.687 |  |  |  | ? | x |
|  | 6 | 3.423 | x | x |  | x | x |
|  | 7 | 2.966 | x |  | x | x | x |
|  | 8 | 2.223 | ? | x |  |  |  |
|  | 9 | 4.605 | x |  | x | x | x |
|  | 10 | 1.236 |  |  |  | x | x |
| CAGE 15-2 893B 0-1 cm - Specimen 12 | 1 | 3.031 |  |  |  | x | x |
|  | 2 | 2.232 |  |  |  | x | x |
|  | 3 | 2.938 | x |  | x | x | x |
|  | 4 | 1.845 |  | x | x | x | ? |
|  | 5 | 1.879 | x |  | x | x | x |
|  | 6 | 2.866 | x |  | x | x | x |
|  | 7 | 2.273 |  |  |  | x | x |
|  | 8 | 2.387 |  |  |  | x | x |
|  | 9 | 1.310 |  |  |  | x | x |
|  | 10 | 1.405 |  |  |  | x | x |
| CAGE 15-2 893B 0-1 cm - Specimen 13 | 1 | 2.156 |  |  |  | x | x |
|  | 2 | 2.368 | x |  | x | x | x |
|  | 3 | 2.473 |  |  |  | x | x |
|  | 4 | 1.795 |  |  |  | x | x |
|  | 5 | 3.626 |  |  |  | x | x |
|  | 6 | 3.613 |  |  |  | x | x |
|  | 7 | 2.515 |  |  |  | x | x |
|  | 8 | 2.322 |  |  |  | x | x |
|  | 9 | 1.849 |  |  |  | x | x |
|  | 10 | 4.116 |  |  |  | x | x |
| CAGE 15-2 893B 0-1 cm - Specimen 14 | 1 | 2.405 | x |  |  | x | x |
|  | 2 | 2.470 |  |  |  | x | x |
|  | 3 | 2.271 | x |  |  | x | x |
|  | 4 | 2.381 |  |  |  | x | x |
|  | 5 | 5.445 | x |  | x | x | x |
|  | 6 | 2.190 |  |  |  | x | x |
|  | 7 | 1.450 |  |  |  | x | x |
|  | 8 | 1.990 | ? |  |  | x | x |
|  | 9 | 1.450 | n/a | n/a | n/a | n/a | n/a |
|  | 10 | 1.978 |  |  |  | x | x |
| CAGE 15-2 893B 0-1 cm - Specimen 15 | 1 | 2.310 | x |  | x | x | x |
|  | 2 | 3.015 |  | x |  |  |  |
|  | 3 | 4.420 |  |  |  | x | x |
|  | 4 | 2.439 |  |  |  | x | x |
|  | 5 | 3.653 | x |  | x | x | x |
|  | 6 | 2.722 |  |  |  | x | x |
|  | 7 | 0.764 |  | x |  |  |  |
|  | 8 | 1.732 |  |  |  | x | x |
|  | 9 | 1.220 |  | x | x | x | x |
| CAGE 15-2 893B 1-2 cm - Specimen 16 | 1 | 1.958 |  |  |  | x | x |
|  | 2 | 1.854 |  |  |  | x | x |
|  | 3 | 1.689 |  |  |  | x | x |
|  | 4 | 3.392 |  |  |  | x | x |
|  | 5 | 1.721 |  |  |  | x | x |
|  | 6 | 2.832 | x |  | x | x | x |
|  | 7 | 1.421 |  | x | x | x | ? |
|  | 8 | 1.184 | x |  | x | x | x |
|  | 9 | 1.590 |  |  |  | x | x |
|  |  |  |  |  |  |  |  |
| CAGE 15-2 893B 1-2 cm - Specimen 17 | 1 | 3.858 | x |  | x | x | x |
|  | 2 | 2.590 |  |  |  | x | x |
|  | 3 | 1.881 |  |  |  |  | x |
|  | 4 | 2.532 |  |  |  | x | x |
|  | 5 | 3.814 | x |  | x | ? | x |
|  | 6 | 3.174 |  |  |  | x | x |
|  | 7 | 2.233 |  | x |  | x | x |
|  | 8 | 2.852 |  | ? |  | x | x |
|  | 9 | 2.931 |  |  |  | x | x |
|  | 10 | 3.718 | x | ? | x | x | x |
|  | 11 | 2.013 | x | x |  | x |  |
|  | 12 | 2.174 | x |  | x | x | x |
| CAGE 15-2 893B 1-2 cm - Specimen 18 | 1 | 2.122 |  |  |  | x | x |
|  | 2 | 2.822 | x | x | ? | x | ? |
|  | 3 | 2.497 | x | x | x | x | ? |
|  | 4 | 3.192 |  | x |  | x | x |
|  | 5 | 1.920 | x |  |  |  |  |
|  | 6 | 3.849 | n/a | n/a | n/a | n/a | n/a |
|  | 7 | 2.172 |  |  |  | x | x |
|  | 8 | 1.597 |  |  |  | x | x |
|  | 9 | 1.291 |  | x | x | x | ? |
| CAGE 15-2 893B 1-2 cm - Specimen 19 | 1 | 2.556 | n/a | n/a | n/a | n/a | n/a |
|  | 2 | 2.724 |  |  |  | ? | x |
|  | 3 | 2.987 | x |  | x | x | x |
|  | 4 | 2.229 |  | ? |  | x | x |
|  | 5 | 1.728 |  | x |  | x |  |
|  | 6 | 1.306 |  | ? | x | x | x |
|  | 7 | 3.335 | x |  | x | x | x |
|  |  |  |  |  |  |  |  |
|  |  |  |  |  |  |  |  |
| CAGE 15-2 893B 1-2 cm - Specimen 20 | 1 | 1.535 |  |  |  | x | x |
|  | 2 | 2.549 |  |  |  | x | x |
|  | 3 | 1.142 | x |  |  | x | x |
|  | 4 | 1.239 | ? | x |  | x |  |
|  | 5 | 4.403 |  |  |  | x | x |
|  | 6 | 6.100 |  |  |  | x | x |
|  | 7 | 1.024 | x |  | x |  | x |
|  | 8 | 1.461 | x | ? | x | x | x |
| CAGE 15-2 893B 1-2 cm - Specimen 21 | 1 | 2.329 | n/a | n/a | n/a | n/a | n/a |
|  | 2 | 2.679 | x |  | x | x | x |
|  | 3 | 2.549 | x |  | x | x | x |
|  | 4 | 2.852 | x |  | ? | ? | x |
|  | 5 | 2.470 | x |  | x | x | x |
|  | 6 | 1.302 | x | x | x | x | x |
|  | 7 | 3.022 | x | ? |  | x | x |
|  | 8 | 1.718 |  |  |  | x | x |
|  | 9 | 2.171 |  |  | x | x | x |
|  | 10 | 2.023 | x |  | x | x | x |
|  | 11 | 1.925 |  |  |  | x | x |
|  | 12 | 3.716 | x |  | x | x | x |
|  | 13 | 2.426 |  |  |  | x | x |
|  | 14 | 3.247 | x |  | x | x | x |
|  | 15 | 2.017 | n/a | n/a | n/a | n/a | n/a |
| CAGE 15-2 893B 1-2 cm - Specimen 22 | 1 | 2.355 |  |  |  | x | x |
|  | 2 | 2.779 |  |  |  | x | x |
|  | 3 | 6.256 |  |  |  | x | x |
|  | 4 | 2.510 |  |  |  | x | x |
|  | 5 | 2.530 | x |  |  | ? | x |
|  | 6 | 3.899 |  |  |  | x | x |
|  | 7 | 1.879 |  | x | x | x | x |
|  | 8 | 2.079 | x |  | x | x | x |
|  | 9 | 2.355 | x | x | x | x | x |
|  | 10 | 1.676 |  | ? |  | x | x |
| CAGE 15-2 893B 1-2 cm - Specimen 23 | 1 | 3.556 |  |  |  | x | x |
|  | 2 | 5.263 |  |  |  | x | x |
|  | 3 | 1.562 | n/a | n/a | n/a | n/a | n/a |
|  | 4 | 1.910 | x |  | x | x | x |
|  | 5 | 2.466 |  | ? |  |  | ? |
|  | 6 | 3.733 |  |  |  | x | x |
|  | 7 | 4.928 |  |  |  | x | x |
|  | 8 | 1.380 |  | x |  |  |  |
|  | 9 | 2.856 |  |  |  | x | x |
|  | 10 | 1.372 |  | ? |  | x | x |
| CAGE 15-2 893B 1-2 cm - Specimen 24 | 1 | 5.245 | n/a | n/a | n/a | n/a | n/a |
|  | 2 | 2.822 | n/a | n/a | n/a | n/a | n/a |
|  | 3 | 4.149 | n/a | n/a | n/a | n/a | n/a |
|  | 4 | 2.646 |  |  |  | x | x |
|  | 5 | 1.921 |  |  |  | x | x |
|  | 6 | 2.406 | x |  |  | x | x |
|  | 7 | 2.409 | x |  | x | x | x |
|  | 8 | 2.262 |  |  |  | x | x |
|  | 9 | 4.048 | n/a | n/a | n/a | n/a | n/a |
|  | 10 | 2.017 | x |  |  | x | x |
|  | 11 | 1.163 |  |  |  | x | x |
|  | 12 | 4.426 | n/a | n/a | n/a | n/a | n/a |
|  | 13 | 2.513 | x |  |  | x | x |
|  | 14 | 1.278 |  | x |  | x | x |
|  | 15 | 2.095 | n/a | n/a | n/a | n/a | n/a |
|  |  |  |  |  |  |  |  |
|  |  |  |  |  |  |  |  |
| HH13 000BC 1-2 cm - Specimen 12 | 1 | 1.864 |  |  |  | x | x |
|  | 2 | 2.426 |  |  |  | x | x |
|  | 3 | 2.427 | ? |  | x | ? | x |
|  | 4 | 1.059 |  | x |  | ? | x |
|  | 5 | 1.878 |  |  |  | x | x |
| HH13 000BC 1-2 cm - Specimen 13 | 1 | 2.949 |  |  |  | x | x |
|  | 2 | 1.195 | x | ? |  | x |  |
|  | 3 | 1.272 |  |  |  | x | ? |
|  | 4 | 1.053 | x | ? |  | x | x |
|  | 5 | 1.140 | x |  |  | x | x |
|  | 6 | 2.302 | x |  | x | x | x |
|  | 7 | 1.776 | x |  | x | ? | x |
|  | 8 | 4.051 |  |  |  | x | x |
| VIB 10 138 72-74 cm - Specimen 2 | 1 | 2.837 |  |  |  | x | x |
|  | 2 | 4.466 |  |  |  | x | x |
|  | 3 | 4.420 |  |  |  | x | x |
|  | 4 | 1.648 |  | ? |  | x | x |
|  | 5 | 2.023 |  |  |  | x | x |
|  | 6 | 2.531 |  | ? |  | x | x |
|  | 7 | 1.487 |  |  |  | x | x |
|  | 8 | 2.816 | x |  |  | x | x |
|  | 9 | 3.242 |  |  |  | x | x |
|  | 10 | 1.665 |  |  |  | x | x |
|  | 11 | 6.063 |  |  |  | ? | x |
|  | 12 | 1.644 | ? |  |  | x | x |
|  | 13 | 2.445 |  |  |  | x | x |
|  | 14 | 2.160 |  |  | x | x | x |
|  | 15 | 4.366 | x |  | x | x | x |
|  |  |  |  |  |  |  |  |
|  |  |  |  |  |  |  |  |
| VIB 10 138 72-74 cm - Specimen 3 | 1 | 2.419 |  |  |  | x | x |
|  | 2 | 4.040 | ? |  |  | x | x |
|  | 3 | 3.310 |  |  |  | x | x |
|  | 4 | 3.011 |  |  |  | x | x |
|  | 5 | 2.675 | n/a | n/a | n/a | n/a | n/a |
|  | 6 | 2.228 |  | ? |  | x | x |
|  | 7 | 2.131 |  | ? | ? | x | ? |
|  | 8 | 1.795 |  |  | ? | x | x |
|  | 9 | 2.129 |  |  |  | x | x |
|  | 10 | 2.782 |  |  |  | x | x |
|  | 11 | 1.158 |  |  |  | x | x |
|  | 12 | 2.492 |  |  |  | x | x |
|  | 13 | 2.041 | ? | ? | ? | x | x |
| VIB 10 138 72-74 cm - Specimen 4 | 1 | 2.388 |  |  |  | x | x |
|  | 2 | 7.817 |  |  |  | x | x |
|  | 3 | 2.399 |  |  |  | ? | x |
|  | 4 | 2.952 |  |  |  | x | x |
|  | 5 | 1.620 |  | ? |  | x | x |
|  | 6 | 2.567 | ? |  |  | x | x |
|  | 7 | 0.867 |  |  |  | x | x |
|  | 8 | 1.938 |  |  | x | x | x |
|  | 9 | 1.523 |  | ? |  | x | x |
|  | 10 | 1.556 | x |  |  |  | x |
|  | 11 | 1.146 |  |  | x | x |  |
|  | 12 | 2.705 | x |  |  |  | x |
|  | 13 | 1.976 |  | ? |  |  |  |
| FR 320 - Specimen 10 | 1 | 3.480 |  |  |  | x | x |
|  | 2 | 3.135 |  |  | x | x | x |
|  | 3 | 4.679 |  |  |  | x | x |
|  | 4 | 1.907 |  |  | x | x | x |
|  | 5 | 1.552 |  |  |  | x | x |
|  | 6 | 2.883 |  |  |  | x | x |
|  | 7 | 4.786 |  |  |  | x | x |
|  | 8 | 1.664 |  |  |  | x | x |
|  | 9 | 3.611 |  |  |  | x | x |
|  | 10 | 3.954 |  |  |  | x | x |
|  | 11 | 2.335 |  |  |  | x | x |
|  | 12 | 1.921 |  |  | x | x | x |
|  | 13 | 1.547 |  |  |  | x | x |
| PC06 Sect5 - Specimen 4 | 1 | 2.389 | n/a | n/a | n/a | n/a | n/a |
|  | 2 | 2.743 | n/a | n/a | n/a | n/a | n/a |
|  | 3 | 2.846 | n/a | n/a | n/a | n/a | n/a |
|  | 4 | 1.994 | n/a | n/a | n/a | n/a | n/a |
|  | 5 | 3.643 | n/a | n/a | n/a | n/a | n/a |
|  | 6 | 3.254 | n/a | n/a | n/a | n/a | n/a |
|  | 7 | 4.162 | n/a | n/a | n/a | n/a | n/a |
|  | 8 | 5.405 | n/a | n/a | n/a | n/a | n/a |

**Supplementary Table S4.** Summary of the energy dispersive x-ray spectroscopy (EDS) and electron backscattered diffraction (EBSD) analytical conditions.

| Energy dispersive x-ray spectroscopy (EDS) | X-max 80 system | Quantax 70 |
| --- | --- | --- |
| Acceleration voltage | 10.0 kV | 15.0 kV |
| Working distance | 8.6 mm | 8.0-8.9 mm |
| Aperture | 60 μm | 150 μm |
| Energy range | 10 keV | 15 keV |
| Process time | 4 |  |
| Map resolution (width) | 1024 pixels |  |
| Map resolution (height) | 768 pixels |  |
| Area (map) | 27.6 x 20.7 μm |  |
| Number of frames (map) | 2 |  |
| Total counts (map) | 893137 |  |
| Acquisition time (points) | 30.0 s | Manually adjusted |

| Electron backscattered diffraction (EBSD) |  |
| --- | --- |
| Acceleration voltage | 20.0 kV |
| Working distance | 20.8 mm |
| Aperture | 240 μm |
| Specimen tilt | 70° |
| EBSD camera binning | 2 x 2 (672 x 512 pixels) |
| EBSD camera exposure time | 20.8 ms |
| Frame averaging | 2 |
| Background | auto |
| Hough resolution | 70 |
| Band detection mode | center |
| Number of bands | 6 |
| Indexing mode | refined accuracy |
| Indexed phases | calcite, albite, anorthite, orthoclase, quartz |

**Supplementary figure S1.** Mineralogical characterization of sedimentary particles within the calcite shell of *Melonis barleeanus*. (a) Electron backscattered diffraction (EBSD) image, which is an overlaid image of backscatter electron image and forescatter electron image showing the phase identification via point analysis (colored crosses). b-f) Identification patterns of each phase shown in (a). Scale bar is 10 μm.

**Supplementary figure S2.** Results of a mixing model between two end-members. One end-member is represented by a hypothetical calcite with δ^18^O=0‰ and oxygen concentration (O)=479600 ppm. The minerals identified in *Melonis barleeanus* through electron backscattered diffraction (EBSD) analysis are used as the second end-members. More specifically: line a, quartz with δ^18^O=18‰ and O=532600 ppm; line b, albite with δ^18^O=8.5‰ and O=486600 ppm; line c, orthoclase with δ^18^O=8.5‰ and O=459900 ppm; and line d, anorthite with δ^18^O=8.5‰ and O=461400 ppm. Lines c and d overlap. δ^18^O values for detrital quartz and feldspar are from^1^, and are reported relative to the standard mean ocean water (SMOW) standard^2^. Wt % = weight per cent.

**References**

1. Savin, S. M. & S. Epstein, The oxygen and hydrogen isotope geochemistry of ocean sediments and shales. *Geochim. Cosmochim. Acta* **34**(1), 43-63, doi:10.1016/0016-7037(70)90150-x (1970).
2. Craig, H. Standard for reporting concentration of deuterium and oxygen-18 in natural waters. *Science* **133**(3467), 1702-1703, doi:10.1126/science.133.3467.1833.
